# Effective Cerebral Connectivity during Silent Speech Reading Revealed by Functional Magnetic Resonance Imaging

**DOI:** 10.1371/journal.pone.0080265

**Published:** 2013-11-21

**Authors:** Ying-Hua Chu, Fa-Hsuan Lin, Yu-Jen Chou, Kevin W.-K. Tsai, Wen-Jui Kuo, Iiro P. Jääskeläinen

**Affiliations:** 1 Institute of Biomedical Engineering, National Taiwan University, Taipei, Taiwan; 2 Department of Biomedical Engineering and Computational Science, Aalto University School of Science, Espoo, Finland; 3 Institute of Neuroscience, National Yang-Ming University, Taipei, Taiwan; University of Maryland, United States of America

## Abstract

Seeing the articulatory gestures of the speaker (“speech reading”) enhances speech perception especially in noisy conditions. Recent neuroimaging studies tentatively suggest that speech reading activates speech motor system, which then influences superior-posterior temporal lobe auditory areas *via* an efference copy. Here, nineteen healthy volunteers were presented with silent videoclips of a person articulating Finnish vowels /a/, /i/ (non-targets), and /o/ (targets) during event-related functional magnetic resonance imaging (fMRI). Speech reading significantly activated visual cortex, posterior fusiform gyrus (pFG), posterior superior temporal gyrus and sulcus (pSTG/S), and the speech motor areas, including premotor cortex, parts of the inferior (IFG) and middle (MFG) frontal gyri extending into frontal polar (FP) structures, somatosensory areas, and supramarginal gyrus (SMG). Structural equation modelling (SEM) of these data suggested that information flows first from extrastriate visual cortex to pFS, and from there, in parallel, to pSTG/S and MFG/FP. From pSTG/S information flow continues to IFG or SMG and eventually somatosensory areas. Feedback connectivity was estimated to run from MFG/FP to IFG, and pSTG/S. The direct functional connection from pFG to MFG/FP and feedback connection from MFG/FP to pSTG/S and IFG support the hypothesis of prefrontal speech motor areas influencing auditory speech processing in pSTG/S *via* an efference copy.

## Introduction

Speech perception is not limited to hearing, as seeing the articulatory gestures of a speaker, the lip forms, position of the jaw and the tongue, significantly enhances speech perception especially in noisy conditions [1], [2]. Further, as a phenomenon demonstrating that visual information has access to the auditory system at relatively early sound processing stages, presentation of certain combinations of incongruent phonetic sounds and articulatory gestures can result in illusory third-category phonetic percepts, for instance, visual /ga/ and auditory /ba/ often results in the perception of /da/ [3], especially when the auditory stimulus is degraded or presented in noise [1].

There are a number of previous functional magnetic resonance imaging (fMRI) studies that have mapped brain areas that participate in processing of visual speech (*i.e.*, “speech reading”) and/or in which brain areas speech reading influences auditory speech processing. These studies have suggested that auditory processing is robustly modulated especially in the left hemisphere posterior superior temporal gyrus/sulcus (pSTG/S) [4]–[17]. Evidence from magnetoencephalography (MEG) and electroencephalography (EEG) studies further suggest that audiovisual phonetic interactions occur at ∼100–150 ms from sound onset [18]–[21], with MEG inverse estimates localizing this effect in the posterior superior temporal lobe [18], [20].

In addition to the pSTG/S, activation of the speech motor areas, including the Broca's area in the inferior/lateral aspects of the frontal lobe [5], [8], [11], [12], [14], [22]–[26], the motor cortex [12], [14], [17], [22], [26], somatosensory cortex [12], [14], and parietal cortical areas [11], [22], [25], [26], has been consistently observed in fMRI studies with audiovisual and/or visual speech stimuli. Importantly, the speech motor areas appear not only involved in speech production, but seem to also participate in speech perception (for reviews, see [27]–[31]). Specifically, the superior/posterior aspects of the temporal lobe, which also seem to be the site of audiovisual interactions, have been hypothesized to contain representations mapping “doable” articulations with associated sounds [31], in lieu of the motor theory of speech perception [32], [33].

The potential role of speech motor areas in mediating the effect of visual speech cues on auditory processing was recently suggested by an fMRI study where activity patterns in frontal cortical areas, elicited by illusory /ta/ produced by auditory /pa/ and visual /ka/, were from the outset more similar to the pattern of activity elicited by congruent audiovisual /ta/ than by /pa/ or /ka/, whereas the activity patterns in temporo-parietal areas initially resembled that caused by /pa/ and only at a longer latency became to resemble that elicited by /ta/ [14]. This finding was interpreted as suggesting that there was an efference copy from the speech motor system that shaped phonetic perception at the sensory-cortical level [14]. Further supporting the involvement of speech motor system in speech perception, it was recently observed that enhanced early-latency ∼100 ms electromagnetic activity in the left premotor cortex was associated with successful phonetic categorization [34].

Indeed, it has been suggested that perceptual and cognitive functions are “mapped at the level of multi-focal neural systems rather than specific anatomical sites, giving rise to brain-behavior relationships that are both localized and distributed” [35]. Here, we set forth to study the neural basis of speech reading from a network perspective: in addition to mapping the activated areas, we specifically wished to clarify the orchestration among these areas. In previous studies, inter-regional modulation during perception and cognition has been quantified using two types of connectivity analyses. Functional connectivity [36] analyses are based on estimating temporal correlations among brain loci during behavior/cognitive tasks [37]. Analyses of effective connectivity are, in turn, based on estimation of causal influence/interactions among brain regions during different conditions [38], [39]. Here, we used Structural Equation Modeling (SEM) [40]–[45], which uses data covariance/correlations to reveal both the strength and the direction of information flow between designated brain areas [40] to estimate effective connectivity between the brain areas activated during speech reading. We specifically hypothesized that SEM analysis of event-related fMRI data would reveal both feed-forward connectivity from the visual areas to pSTG/S as well as the frontal cortical speech motor areas and, further, direct feedback connections from the frontal speech motor areas to the pSTG/S.

## Materials and Methods

### Ethics Statement and Subjects

nineteen healthy right-handed volunteers (ten females, ages 20–28) participated in the study. The study protocol was in line with the principles outlined in the Helsinki declaration. The study was approved by the ethics committee of National Taiwan University. A written informed consent was obtained from each of the subjects prior to participation.

### Task and stimuli

**Figure 1:**
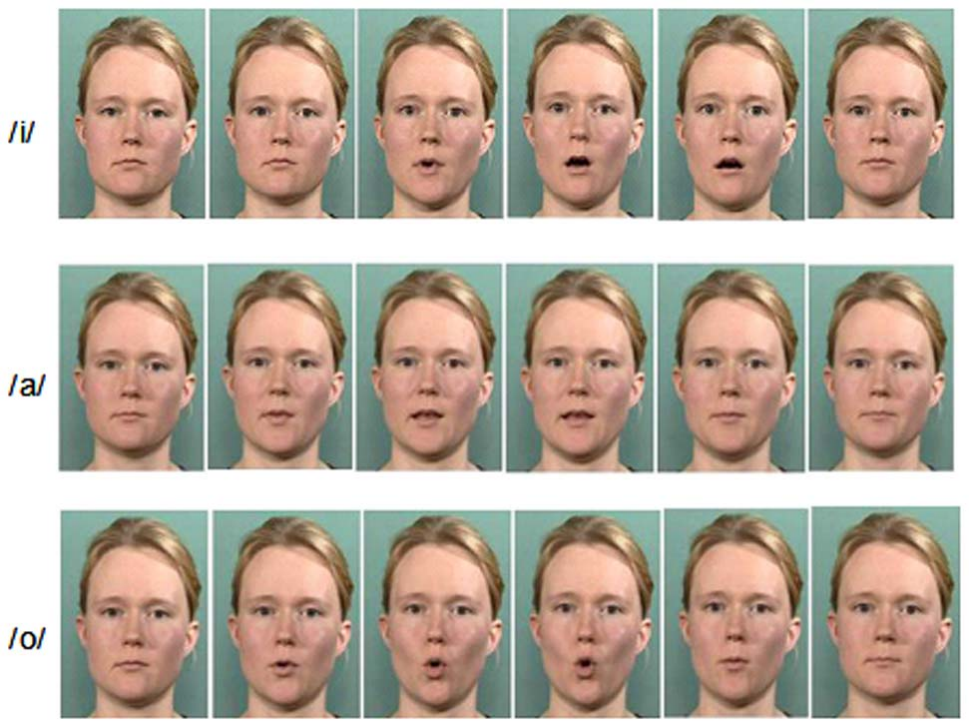
The stimuli that were used in the present study. Shown are individual frames, from the fifth frame with steps of five frames to the 30^th^ frame, from the videoclips of /i/, /a/, and /o/.

The subjects were shown short silent videoclips (24 frames per sec, clip length = 1250 ms) of a female face articulating silently /a/, /i/, and /o/ (see **Figure 1**; the person pictured articulating has given written informed consent to publication of these photographs), with the task of the subjects being to press a button as quickly as possible using their right-hand index finger whenever they detected an /o/ articulation. A total of 42 videoclips of each articulation type were shown to the subjects during the experiment with an inter-stimulus interval (ISI) of 5.5 sec in average (minimum ISI was 3 sec), divided into four runs of four minutes each. Stimuli were distributed randomly and evenly (i.e., a fixed number of stimuli of each type occurring within a single run) across 4 runs. The onsets of the stimuli were optimized in order to obtain the highest efficiency for event-related fMRI [46].

### fMRI data acquisition

3T scanner (Tim Trio, Siemens Medical Solutions, Erlangen Germany) was used to acquire the event-related fMRI data. The fMRI acquisition parameters were: TR/TE = 2000/30 ms, field-of-view (FOV) = 220×220 mm, matrix = 64×64, slice thickness = 4 mm, flip angle = 90°. For each subject, thirty-four trans-axial slices with no gap were acquired with the spatial coverage of cerebrum and cerebellum.

### Data analysis

EPI fMRI data were first pre-processed for motion correction, slice timing correction, coregistration, and spatial normalization using SPM5 software (Wellcome Department of Imaging Neuroscience, London, UK). Functionally active areas were identified using General Linear Model (GLM) to reveal voxels with statistically significant correlation between the measured EPI time series and the modeled hemodynamic response, which was calculated as the convolution between the train of stimulus onsets and a canonical hemodynamic response function (HRF) [47]. Additional confounding effects, such as a DC shifts, linear drifting, and low frequency oscillations over time, were also added to the design matrix of the GLM as regressors describing individual nuisance effects. The hemodynamic responses were estimated for /a/, /i/, and /o/ individually. Second-level random effect analysis was used for the group-level fMRI data analysis. Finally, activated brain areas were identified after statistical thresholding with at *p*<0.05 corrected for multiple comparisons based on the Gaussian Random Field theory [48].

The SEM effective connectivity analysis was conducted as follows: first, EPI time courses from each functionally active area were extracted. At each region, hemodynamic response time courses for each stimulus category (/a/, /i/, and /o/) were estimated by GLM as described above in order to residualize the within-subject variation due to multiple observations [49]. These hemodynamic responses were then used to calculate a data covariance matrix for each subject. Finally the data covariance matrices from all subjects were averaged to generate the data covariance matrix **S**. Here we specifically derived two data covariance matrices for vowels /o/ (target) and /i/+/a/ (non-targets), where the time sources for /i/ and /a/ were averaged. Second, based on existing knowledge, we constructed a model with directional connectivity. A numerical solver was then used to minimize the cost function given the network topology and empirical data. Here, the cost function was quantitatively described by the maximal likelihood (ML) estimator, which targeted on minimizing the cost *F_ML_*:
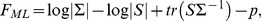
(1)where |.| denotes the determinant of a matrix, tr(.) is the trace of a matrix, and *p* is the number of regions. **S** and Σ are the observed and model implied covariance matrices respectively.
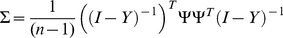
(2)
**Y** is the matrix containing all the path coefficients to be estimated. The element *y_ij_* in the matrix **Y** indicates the causal modulation from area *j* to area *i*. Ψ is the residual covariance matrix describing the variance/covariance not accounted by the paths. *n* is the number of time point in the EPI time series. A smaller *F_ML_* value means the model implied covariance matrix Σ is more similar to the observed covariance matrix **S**. SEM analysis was implemented in Matlab (Mathworks, Natick, MA, USA) using the optimization toolbox. Path coefficients were separately estimated for responses elicited by /o/ and /a/+/i/ stimuli. The statistical inference for each path coefficient was calculated by bootstrap, where 19 subjects were randomly selected with replacement for 100 times to calculate the path coefficients. The significance of a path was non-parametrically tested using the Wilcoxon signed rank test for zero median. The difference between path coefficients in target and non-target conditions was tested using the Wilcoxon rank sum test.

In the SEM analysis of the effective connectivity, as described previously, a directionally connected model must be specified before the path coefficient estimation. Such an *a priori* model changes neither the number of directional paths nor the directionality of any path during path coefficient estimation. Inaccurate specification of the directionally connected model can therefore provide erroneous path estimates. Modifying the directional connections by automatically increasing one or more directional connections can be used to test if a given model is sufficiently accurate or whether an additional directional path could be included to further improve the fitting between **S** and Σ significantly [50]. Here, modification index (MI) was used to estimate how much better Σ fits to **S** when a path not originally included in the model is added (*i.e.*, when the path coefficient is unconstrained from zero [51]. MI was calculated for every directional path not originally included in the model, with the path showing the largest MI included in the model. The MI of the *l* ^th^ path is:
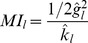
(3)where 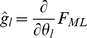 and 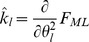. The first order and second order partial derivatives can be approximated as
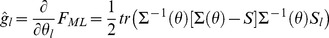
(4)

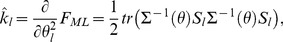
(5)where 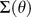 is the model inferred covariance matrix calculated from the parameters **Y** and Ψ. Here we used variable θ to jointly represent the estimated path coefficients **Y** and the covariance of the residual vector Ψ. θ = [vec(**Y**) vec(Ψ)]^T^. The matrix S*_l_* comprises the partial derivatives of the modeled covariance Σ with respect to the *l* ^th^ path from region “A” to region “B” (**Y**_BA_). Such a path was not originally included in the SEM connectivity. S*_l_* is defined as
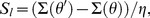
(6)where θ ′ = θ+**e** *_l_*, **e** *_l_* denotes a vector with all entries of zero except the entry corresponding to the *l* ^th^ path is set to one. And *η* is an arbitrarily small constant. We set *η* = 10^−4^ in this study. We iteratively changed the path *l* and calculate the MI*_l_* to respectively test all possible improvements in the SEM by adding one path not originally included in the model. The path with the largest MI*_l_* was added to generate a new directionally connected model and another SEM analysis was done to estimate the path coefficients.

## Results

### Cortical areas activated during lipreading

**Figure 2:**
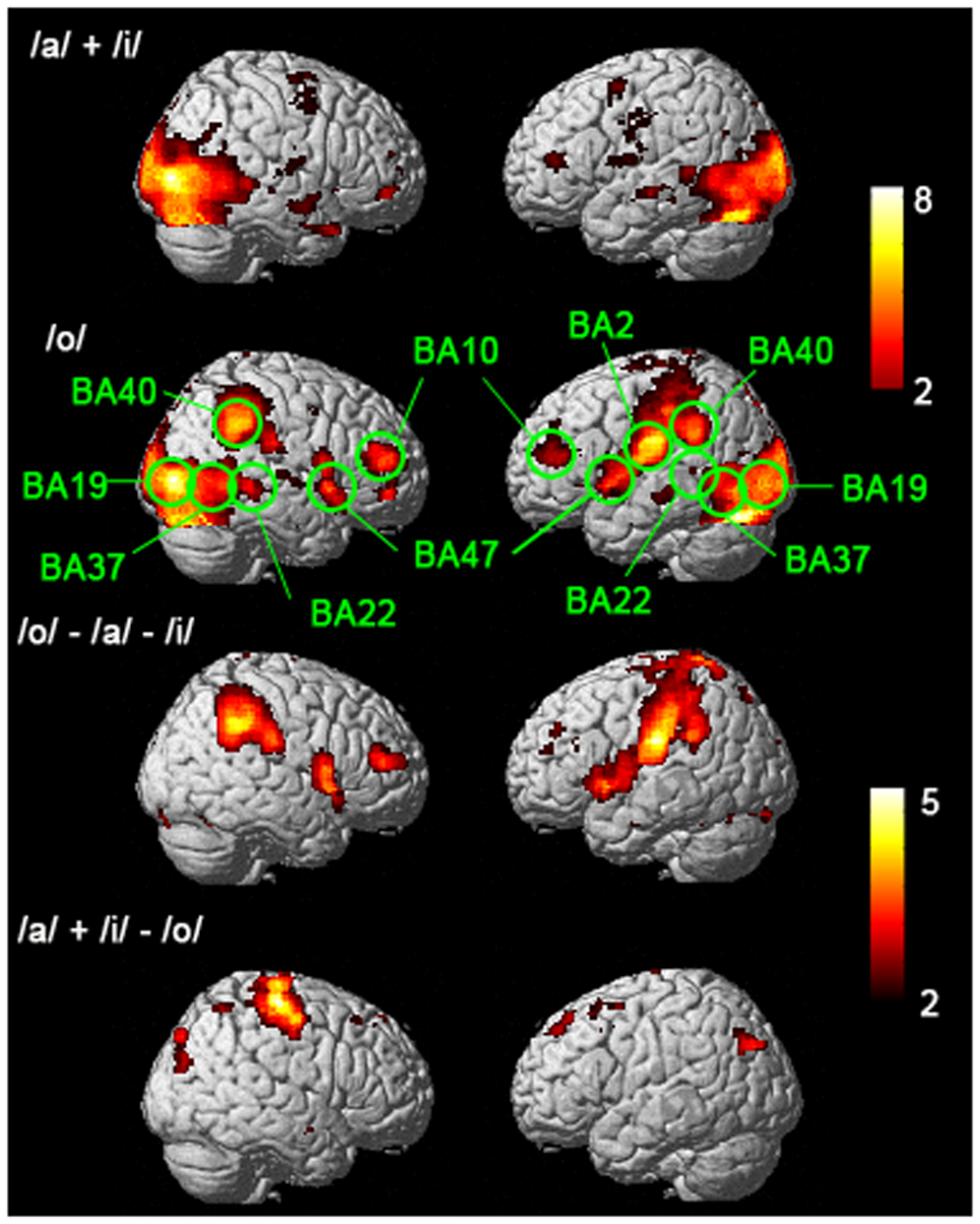
Brain areas significantly activated by viewing videoclips of face articulating non-target (/a/ and /i/) and target (/o/) vowels. The third row shows brain areas that were significantly more active in the target condition than in the non-target condition, and the fourth row the areas that were significantly more active in the non-target condition than the target condition, respectively. Color corresponds to the *t* statistics.

The brain areas with statistically significant event-related fMRI hemodynamic responses to the target (/o/) and non-target (/a/ and /i/) stimuli are shown in **Figure 2**. Their Talairach coordinates and the corresponding Brodmann Areas (BA) are listed in **Table 1**. During perception of the non-target (/a/ and /i/) there were significant bilateral activations, in addition to the obvious visual cortical activations, in the pSTG/S (BA 22), and inferior frontal gyrus (IFG) activations within the classically defined Broca's area (BA 47). Further, there was significant activation in the frontal polar (FP) area (BA 10). In the target condition /o/, significant parietal cortical activity was observed in the secondary somatosensory cortex (BA 2) and in the supramarginal gyrus (SMG) (BA 40). Significant activation of the posterior inferior temporal/fusiform gyrus (pFG) was also observed in the left hemisphere (BA 37). The activations to targets also tended to be somewhat stronger, with the significant activity encompassing larger areas in bilateral BA 10 and BA 47.

**Table 1:** The Tailarach coordinates of activated brain areas, Brodmann Area (BA), t statistics, and corrected *p*-values in target (/o/) and non-target (/a/+/i/) conditions.

Area	X (mm)	Y (mm)	Z (mm)	BA	*t* statistics		*p*-value	
					/a/+/i/	/o/	/a/+/i/	/o/
**1**	60	−40	46	40	1.77	6.33	0.040	0.000
**2**	42	−86	6	19	7.73	7.05	0.000	0.000
**3**	60	−33	3	22	3.49	3.61	0.000	0.000
**4**	50	−50	0	37	6.07	5.53	0.000	0.000
**5**	36	42	17	10	2.5	4.5	0.007	0.000
**6**	48	16	−4	47	1.32	3.4	0.092	0.001
**7**	−42	−80	6	19	4.92	4.04	0.000	0.000
**8**	−60	−17	24	2	1.68	6.33	0.047	0.000
**9**	−49	−59	0	37	5.79	5.02	0.000	0.000
**10**	−50	−46	27	40	2.49	4.59	0.007	0.000
**11**	−37	−50	18	22	3.26	3.6	0.001	0.000
**12**	−40	42	15	10	3.16	3.54	0.001	0.000
**13**	−44	14	−4	47	0.44	3.15	0.281	0.001

### SEM analysis of effective connectivity

**Figure 3:**
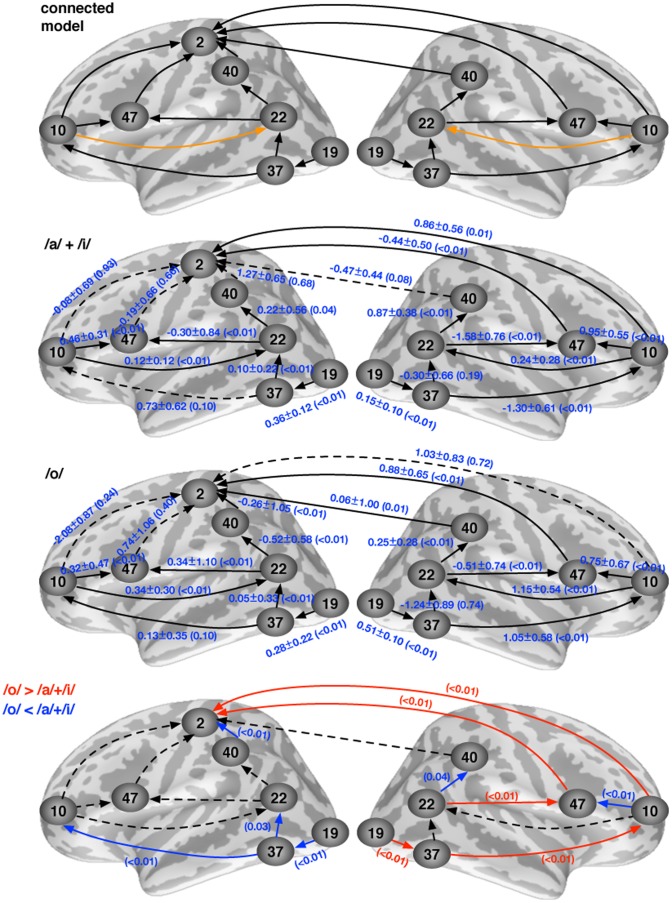
Top panel: the directionally connected model for the SEM analysis on the effective connectivity during speech perception. Note that a path from BA10→BA 22 was added because of this path loaded with the highest MI value (for details, see Materials and Methods). Middle panels: the estimated path coefficients, the standard errors of the mean (SEM), and the associated p-values (in parenthesis) in the /a/+/i/ non-target condition and the /o/ target condition. Statistically significant (*p*≤0.05) and insignificant (*p*>0.05) paths were rendered in solid and dashed lines, respectively. Bottom panel: pair-wise comparison of the path coefficients between target /o/ and non-target /a/+/i/ conditions. Statistically significantly (*p*≤0.05) and insignificantly (p>0.05) different paths were rendered in solid and dashed lines, respectively.

Our directionally connected model suggested that information flow started from the extrastriate visual cortex (BA19), which hierarchically sent information to posterior inferior temporal/fusiform gyrus (BA 37) along the ventral pathway. From BA 37 information was then sent directly to both pSTG/S (BA22) and the MFG/FP (BA 10). The feed-forward model suggested that the information flow continued from BA22 to bi-lateral SMG (BA40), and eventually to somatosensory cortex (BA2). Parallel to this, there were top-down modulations from the MFG/FP (BA 10) to IFG (BA 47), which also received feed-forward information from the pSTG/S (BA 22). From the MFG/FP (BA 10) information was additionally sent to BA 2, which also received information from MFG/FP (BA 10). Note that, except BA 2 in the left hemisphere, all other ROIs were bihemispheric. We used the modification index (MI) to study how to further improve the proposed model. The path with the largest MI and most significantly improving the fit between the observed **S** and empirical Σ data covariance matrices was one running from the MFG/FP (BA10) to the pSTG/S (BA 22). This path was added to the model and the SEM. The SEM model with all specified feed-forward and feedback directional connections is shown in **Figure 3** **(top panel)**.

Two middle panels of **Figure 3** shows the grand average, standard error of the mean (SEM) of the estimated path coefficients, and associated *p* values in non-target /a/ and /i/ condition and target /o/ condition. The SEM was estimated from 100 bootstrap samples. First, the connected model fitted the data reasonably (the non-target condition: χ^2^ = 431, degree of freedom = 1349 (71 free model parameters×19 subjects), *p*>0.99 *H*_0_: data fits SEM model; the target condition: χ^2^ = 480, degree of freedom = 1349, *p*>0.99 *H*_0_: data fits SEM model). The path coefficient can be considered as the amount of the fMRI signal change at the destination ROI given one unit increase of the fMRI signal change in the source ROI. In the non-target condition, most paths were statistically significant (the critical threshold at *p*<0.05), except BA 37 (LH)→BA 10 (LH), BA 47 (LH)→BA 2 (LH), BA 40 (LH)→BA 2 (LH), BA 10 (LH)→BA 2 (LH), and BA 40 (RH)→BA 2 (LH). Here (LH) and (RH) indicate the ROI in left and right hemisphere, respectively. In the target condition, only three paths were insignificant : BA 47 (LH)→BA 2 (LH), BA 10 (LH)→BA 2 (LH), and BA 10 (RH)→BA 10 (LH). The paths suggested by MI (BA 10 (LH)→BA 22 (LH) and BA 10 (RH)→BA 22 (RH)) were all significant in both conditions.

The bottom panel of **Figure 3** shows the comparison between the /a/+/i/ non-target condition and the /o/ target condition. Overall, differential causal modulations in target and non-target conditions were observed. Interestingly, comparing between target and non-target conditions, a few causal modulations stronger in the non-target condition were found lateralized in the left hemisphere (BA 19 (LH)→BA 37 (LH), BA 37 (LH)→BA 23 (LH), BA 37 (LH)→BA 10 (LH), and BA 40 (LH)→BA 2 (LH)). Significant feedback connections (BA 47 (RH)→BA 2 (LH) and BA 10 (RH)→BA 2 (LH)) were found stronger in the non-target condition than in the target condition.

## Discussion

Corroborating findings from previous fMRI [5] and MEG [22] studies, we observed, in addition to visual cortical areas, significant event-related hemodynamic responses during speech reading in the pSTG and in the speech motor areas, including the Broca's area, somatosensory cortex, and parietal cortical areas (see **Figures 2**; **Table 1**). Overall, the responses tended to be stronger for the targets than for non-targets, yet each of these areas were significantly activated also by the non-targets, suggesting that the frontal cortical activations were not due to the motor response task.

The activations that we observed in the IFG (BA 47) and MFG/FP (BA10) are within the limits of the relatively considerable variation in the functional anatomy of frontal-lobe speech production areas [52]. Further, in a recent study, activations specific to audiovisual phonetic level congruency, suggesting the presence of modality-independent phonetic representations, were observed in prefrontal cortical areas extending from IFG to MFG and close to FP [53]. Taken together, these results suggest that the IFG and MFG/FP activations we observed in the present study can be considered to be a part of the frontal-cortical speech motor areas.

Given that we did not have a non-speech biological motion control condition in the present study, it can also be asked to what extent our findings are speech-specific. However, it has been previously documented that the frontal-lobe speech motor system activations are not elicited by lower-face non-speech gestures (*i.e.*, “gurning”), and that the pSTG/S is to a lesser extent activated by gurning than by speech reading [5]. Consequently, it is likely that the presently observed speech motor area and pSTG/S activations during speech reading were at least partly speech specific. In contrast, gurning has been observed to activate pFG (BA 37) more strongly than speech reading [5], and thus it is possible that activation of this area in our study was not speech-specific. In future studies, this should be addressed with both non-speech biological control stimuli and speech-in-noise lip-reading tasks with auditory stimuli.

As a novel approach in the current study, we applied SEM [40]–[45], [50] to the event-related fMRI data, to reveal effective connectivity between the activated brain areas during silent speech reading. Specifically, we used SEM to estimate the strength and direction of information flow between the activated areas (see **Figures 3**). While certain aspects of the feed-forward model corroborated quite well previous estimates of information flow, there were also novel observations. Using MEG, it has been previously estimated that information flows during speech reading from the visual cortical areas to pSTG/S, and then through parietal cortical areas to prefrontal/inferior frontal areas and finally to motor cortex [22]. Our directionally connected model partly agrees with the early aspects of this serial model of information flow, as extrastriate visual cortex (BA 19) was estimated to feed information to pFG (BA 37) along the ventral pathway, and from pFG information flow was estimated to continue to pSTG/S, and on to supramarginal gyrus, and eventually somatosensory cortex (see **Figure 3**). However, our connectivity analysis additionally revealed parallel feed-forward inputs, suggesting that from pFG information flows directly to MFG/FP (BA 10).

Furthermore, direct effective connections from pSTG/S to IFG were suggested by our SEM modelling. It is possible that the differences between the findings in our study and the previous MEG observations [22] are explained by two factors: still rather than dynamic visual stimuli were used in the previous MEG study, which has been observed to affect fMRI activations [23], and the MEG source analysis is always subject to some degree of localization uncertainty due to the ill-posed nature of the inverse problem [54]. Additionally, caution must be exercised with respect to reaching conclusions based on the negative findings (*i.e.*, not seeing a significant effect). However, this cautionary note does not hold for the findings that were statistically significant. Naturally, one should always be careful when comparing fMRI and MEG results because of potential factors explaining differences between our fMRI study and previous MEG studies including differences in what is measured (hemodynamics *vs.* synchrony of neural post-synaptic potentials) and the temporal accuracy of the methods (seconds *vs.* milliseconds).

Notably, our SEM model suggested feedback connectivity in addition to the feedforward connectivity. From the MFG/FP (BA 10), parallel feedback connections were suggested to run to sensorimotor areas (BA 2) in the vicinity of the motor strip representations of the lips, to the adjacent IFG areas, and to pSTG/S. This latter connection is especially noteworthy, given recent findings suggesting that speech motor area activation during speech reading modulates speech processing in superior-posterior temporal lobe areas *via* sending a feedback efference copy [14]. It has to be noted, however, that in this previous study, the anatomical loci of the speech motor and sensory processing areas were somewhat more posterior than the MFG/FP and pSTG/S that our results suggest to be effectively connected. Potential explanations for these discrepancies include differences in functional anatomy between the subject populations, and differences in the type of stimuli that were used (*i.e.*, visual only *vs.* audiovisual, vowels *vs.* consonant-vowel syllables).

The feedback connection from MFG/FP to pSTG/S that we observed also adds to the growing pool of evidence suggesting that the speech motor cortical activations during speech reading are not merely epiphenomena due to the subjects covertly self-articulating, but rather that the inputs from the frontal cortex shape processing within the pSTG/S [14]. Further, the fact that the effective feedback connections were estimated to run from MFG/FP, rather than from the motor cortex, to pSTG/S tentatively suggest that the activation of higher-order language representations plays a more central role in sending the efference copy than the activation of, for instance, the tongue representations in the motor cortex, which has been also implicated to play a role in shaping auditory processing [55]. Interestingly, this effective connectivity match the anatomical connectivity in macaque monkeys: efferent connections from the BA 10 travel down to superior temporal sulcus, extending into the posterior division of the superior temporal sulcus [56]. It is possible that this corresponds to the BA 22 activation that we observed, considering the anatomical differences between monkey and human.

Naturally, in order for the speech motor system to send modulatory feedback inputs, the frontal cortical activations would have to occur at relatively short latencies. In the present study, the MFG/FP areas were estimated to receive direct feed-forward connections from the pFG, suggesting that visual information could have relatively fast access to prefrontal phonological representations. Tentatively, combined MEG/fMRI findings have suggested that visual information can quickly access object representations in the orbital frontal cortex *via* the dorsal stream that then send feedback inputs (“initial guesses”) to shape object processing in the ventral processing stream [57]. Further, in a recent MEG study, a short-latency IFG response was observed during visual word presentation [58] and when listening to auditory syllables [34]. Thus, based on these MEG findings, it seems possible that visual information can access quickly the frontal cortical areas, allowing for feedback modulation of auditory processing within the time frame in which audiovisual interactions have been reported to occur [59].

The primary reason for using an active task in the present study was to make certain that the subjects were attentively lipreading the articulations as instructed. However, we analyzed the BOLD responses and effective connectivity during targets and non-targets separately given that there is extensive literature that has documented differential responses to visual target and non-target stimuli [60], [61]. The demands posed to the subjects by this task during target and non-target trials are holding target information in working memory and matching the information to incoming stimuli *via* the process of sensory discrimination. The target and non-target trials differed, however, in that for the targets the subjects needed to produce a motor response, and for the non-targets the subjects needed to inhibit the response. Indeed, as can be seen in Figure 2, in addition to obvious motor cortical responses for targets, there was differential pattern of activity observed in prefrontal and parietal cortical areas during processing of both targets and non-targets, possibly reflecting demands for production and inhibition of the response.

Effective connectivity also differed significantly during processing of targets *vs.* non-targets, as shown in the bottom panel of **Figure 3**. Interestingly, some inter-hemispheric differences emerged in this analysis with right-hemispheric advantage during processing of target articulations observed for the feed-forward information flow from BA19 to BA37 and on to BA10. In contrast, left-hemisphere advantage along this feed-forward pathway was noted during non-target articulations. Given the role of right hemisphere in selective attention one could, tentatively, assume that the stronger right-hemisphere feed-forward connectivity from visual cortex observed for targets could reflect re-shaping of visual cortical processing by selective attention to filter the sensory features of the target stimulus, as has been shown to take place in higher-order visual areas of monkeys [62], [63] and primary auditory cortex of ferrets [64]. During target processing feedback connections from right-hemisphere BA47 and BA10 on to left-hemisphere BA2 were also pronounced, however, the right-hemisphere feedback connection from BA10 to BA47 was augmented during processing of non-targets. Overall, given that we only had one target type and target probability in our study, our paradigm was not optimized for teasing out target *vs.* non-target effects. For a recent study demonstrating why these factors should be carefully controlled, see [61]. Thus, these results should be considered as tentative observations that warrant further studies on the neural mechanisms underlying target *vs.* non-target processing of visually presented phonetic articulations.

In conclusion, our SEM results suggest that there are feed-forward connections from the visual areas directly to prefrontal cortical areas, and feedback connections from prefrontal cortical areas to the pSTG/S during silent speech reading. These findings lend support for the hypothesis that visual lipreading information quickly activates prefrontal speech motor areas that send an efference copy to the pSTG/S to modulate speech perception in noise.
