# Correction: Effective Cerebral Connectivity during Silent Speech Reading Revealed by Functional Magnetic Resonance Imaging

**DOI:** 10.1371/journal.pone.0097210

**Published:** 2014-05-02

**Authors:** The PLOS ONE Staff

The rows in Figure 1 are labeled in the incorrect order. Please see the corrected figure below.

**Figure 1:**
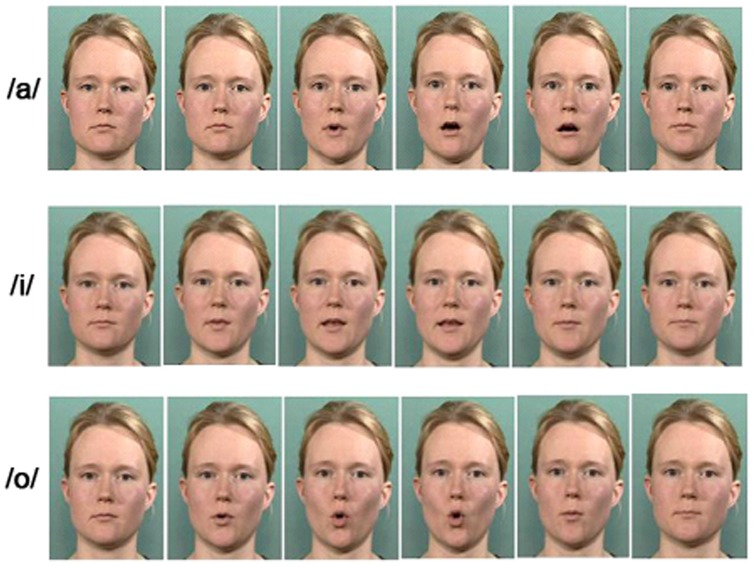
The stimuli used in the present study. Shown are individual frames, from the fifth frame with steps of five frames to the 30th frame, from the videoclips of /a/, /i/, and /o/.
